# Satellite Data-Based Phenological Evaluation of the Nationwide Reforestation of South Korea

**DOI:** 10.1371/journal.pone.0058900

**Published:** 2013-03-08

**Authors:** Su-Jong Jeong, Chang-Hoi Ho, Sung-Deuk Choi, Jinwon Kim, Eun-Ju Lee, Hyeon-Ju Gim

**Affiliations:** 1 Department of Geosciences, Princeton University, Princeton, New Jersey, United States of America; 2 School of Earth and Environmental Sciences, Seoul National University, Seoul, Korea; 3 School of Urban and Environmental Engineering, Ulsan National Institute of Science and Technology, Ulsan, Korea; 4 Department of Meteorology, University of California Los Angeles, Los Angeles, California, United States of America; 5 School of Biological Sciences, Seoul National University, Seoul, Korea; The Ohio State University, United States of America

## Abstract

Through the past 60 years, forests, now of various age classes, have been established in the southern part of the Korean Peninsula through nationwide efforts to reestablish forests since the Korean War (1950–53), during which more than 65% of the nation's forest was destroyed. Careful evaluation of long-term changes in vegetation growth after reforestation is one of the essential steps to ensuring sustainable forest management. This study investigated nationwide variations in vegetation phenology using satellite-based growing season estimates for 1982–2008. The start of the growing season calculated from the normalized difference vegetation index (NDVI) agrees reasonably with the ground-observed first flowering date both temporally (correlation coefficient, *r* = 0.54) and spatially (*r* = 0.64) at the 95% confidence level. Over the entire 27-year period, South Korea, on average, experienced a lengthening of the growing season of 4.5 days decade^−1^, perhaps due to recent global warming. The lengthening of the growing season is attributed mostly to delays in the end of the growing season. The retrieved nationwide growing season data were used to compare the spatial variations in forest biomass carbon density with the time-averaged growing season length for 61 forests. Relatively higher forest biomass carbon density was observed over the regions having a longer growing season, especially for the regions dominated by young (<30 year) forests. These results imply that a lengthening of the growing season related to the ongoing global warming may have positive impacts on carbon sequestration, an important aspect of large-scale forest management for sustainable development.

## Introduction

Most of the forest in South Korea was devastated during the Japanese occupation (1905–1935) and the Korean War (1950–1953). Specifically, forest area in South Korea was reduced to 36–40% of that of the pre-war period [1]. However, with the post-war economic growth, massive nationwide reforestation efforts have been undertaken to reconstruct and manage the nation's forests [1]. To understand the success of the nationwide reforestation effort, previous studies have evaluated changes in forest biomass, area, and growing stock [2], [3], [4]. Although these studies concurrently reported that the nationwide effort has resulted in massive young forests with various and almost continuous ages [2], [3], [4], which now account for 72% of the total forest biomass in South Korea, changes in the seasonal cycle of vegetation growth (e.g., phenology) remain poorly understood. This massive and organized nationwide reforestation effort, unique in the world's history, provides us with an opportunity to investigate the temporal variations in the characteristics of the ecosystem over the course of vegetation growth.

In general, phenology is the seasonal cycle of vegetation growth and/or photosynthetic activity including specific seasonal transition events, such as flowering, budburst, leaf flushing, leaf coloring, and leaf drop [5]. The emergence and growth of leaves in deciduous forests and the beginning of photosynthesis in evergreen forests generally indicate carbon uptake by the forests [6]. Therefore, the observed seasonal cycle of the concentration of atmospheric CO_2_ is attributed to the rhythm of vegetation phenology [7]. It should be noted that phenology has been altered substantially through the last several decades [8], [9]. These changes in plant growing season further contribute to forest productivity and terrestrial carbon exchange [10], [11], [12], [13]. Therefore, in addition to changes in forest biomass related to the nationwide reforestation of Korea, changes in nationwide vegetation phenology and the influences of phenological variations in vegetation on forest growth and/or productivity should be investigated.

Difficulties in analyzing the influence of the growing season of vegetation on the nationwide forest biomass in carbon stock are due to the difficulty of monitoring vegetation phenology over wide areas. Ground observations provide detailed temporal variations for specific species but have limited spatial coverage, which has been a major shortcoming of previous studies of the growing season based on flowering data of a large area, such as the entire nation [14], [15]. Fall phenology data are even more limited. To overcome this weakness of ground observations, some researchers have tried to understand long-term variations in phenology from satellite-retrieved vegetation indices [16], [17]. The satellite-retrieved growing season is generally considered to be the product of a top-down approach. Space observation provides information with wide spatial extent and with vegetation characteristics averaged over multiple plant species, but it provides this information with limited temporal coverage because satellite observations began in 1981. The two aforementioned types of phenology data, ground- and space-based, utilize completely different methodologies for determining spatial and temporal variations in phenology. Thus, a combination of these two types of data would be most useful for measuring variations in surface phenology over a vast area.

To understand the satellite-retrieved phenology in relation with ground observations, this study examined the changes in vegetation growing season over South Korea using the Global Inventory Monitoring and Modeling Studies (GIMMS) normalized difference vegetation index (NDVI) from NOAA satellites and the ground-observed first flowering date (FFD) of cherry. As noted in previous studies, FFD data in South Korea are among the longest historical observations in the world [14], [15]. Despite the scale difference between satellite and ground data, FFD may be appropriate for validating satellite-retrieved surface phenology [19]. The main objectives of this study include (1) retrieving the satellite-based surface phenology, (2) evaluating the temporal and spatial variations in the satellite-retrieved phenology, and (3) evaluating the relationships between spatial variations in surface phenology and forest biomass carbon stocks.

Specifically, we intended to answer the question, “Which phenological event, i.e., start or end of the growing season, has had the most dominant effects on the recent changes in the total growing season?” This is important for understanding regional variations in the growing season as the phenological event most influential in the variations in the total growing season may vary with region and time [8]. In addition, we examined whether forest age affects the relationship between phenology and biomass carbon stocks by making use of the nationwide, post-Korean War reforestation, which resulted in various types of forest with various age compositions [1]. Tree-age compositions are randomly distributed over the nation due to province-based management policies, indicating apparent spatial variations in forest biomass carbon [3]. An evaluation of relationships between spatial variations in the surface growing season and forest biomass carbon will help in the preparation of a new forest management plan for mitigating climate change.

## Methodology

### GIMMS NDVI data

The contrast between the visible and near-infrared (NIR) reflectance of the land surface is closely correlated with the amount of absorbed photosynthetically active radiation and, thus, the amount of biomass of leaves. Photosynthetically active leaves absorb mostly in the visible spectrum and reflect mainly in the NIR spectrum [20], [21]. The NDVI, obtained from this optical property of active leaves, is the most widely used satellite-retrieved vegetation index and has been used for evaluating surface phenology in many previous studies [8], [16], [17], [22]. In this study, NDVI values at spatial and temporal resolutions of 8×8 km and 15 days, interactively, were acquired from the GIMMS group and derived from the Advanced Very High Resolution Radiometer (AVHRR) onboard NOAA satellite series for 1982–2008. The GIMMS NDVI data were corrected to remove several non-vegetation effects caused by aerosols, clouds, volcanoes, sensor degradation, and satellite drift [21].

### First flowering date (FFD) data

To validate satellite-retrieved surface phenology against ground observations, the FFD data of cherry (*Prunus yedoensis*) at 9 stations were obtained from the Korean Meteorological Administration (KMA) data archives (Table 1). KMA has been recording the first fully opened flowers as the FFD of the tree. To alleviate the effects of tree aging, trees in the phenological garden have been replaced at regular intervals of between 15 and 25 years. FFD data from the KMA have been used to address a number of environmental issues (e.g., regional warming [14], [15] and species variations [23]). For more detailed information on FFD, readers are referred to previous studies [14], [15].

**Table 1:** Station information, mean SGS and FFD for the period 1982–2008, and correlation coefficients between SGS and FFD.

Station	Latitude	Longitude	Mean SGS (days)	Mean FFD (days)	Correlation (FFD vs. SGS)
Chuncheon	37.5°N	127.4°E	123.0	102.6	0.61
Wonju	37.2°N	127.5°E	122.9	101.3	0.66
Chungju	36.5°N	127.6°E	119.3	100.0	0.44
Chupungnyeong	36.1°N	128.0°N	116.8	99.4	0.54
Inje	38.0°N	128.1°E	125.7	107.6	0.46
Hongcheon	37.4°N	127.5°E	123.9	104.8	0.43
Boeun	36.3°N	127.4°E	119.7	102.9	0.46
Uiseong	36.2°N	128.4°E	124.6	100.5	0.55
Geochang	35.4°N	127.5°E	124.4	98.1	0.44

### Forest biomass carbon stocks

In this study, we analyzed previously published datasets of forest biomass carbon stock, density, and age composition for 61 forests in South Korea [2], [3]. We now briefly address the characteristics of these data. The forest carbon stock data were obtained by calculating the total tree biomass based on forest-inventory observations for 1954–2000 [24]. In order to convert timber volume into total tree biomass (*M_Tree_*), which includes branches, leaves, and roots, a volume-derived method that requires the specification of the dried wood specific density and the ratios of aboveground tree biomass (timber+branches+leaves) to timber biomass and of total tree biomass (timber+branches+leaves+roots) to aboveground tree biomass was used:

*M_Tree_* = *V*×*D*×*R_a_*×*R_t_*,

where *V* (m^3^) is 3-year averaged tree volume; *D* (Mg m^−3^), the measured dried specific density; *R_a_*, the measured ratio of aboveground tree biomass to timber biomass; and *R_t_*, the measured ratio of total biomass to aboveground tree biomass.

The resulting total tree biomass was converted to carbon mass *C_t_* (Mg) using the ratio of carbon to total tree mass [2],

C_*t*_ = M_*Tree*_×C_*c*_,

This study categorized Korean forests into two types: coniferous and deciduous. The values of *D*, *R_a_*, and *R_t_* for the two forest types are from the values of Korean Pine and Oriental Chestnut Oak [1], [25]. A value of 0.5 was used for *C_c_*, as recommended by the Intergovernmental Panel on Climate Change [26]. Owing to the lack of biomass expansion factors for the various forest types in Korea, previous studies assumed that Korean Pine and Oriental Chestnut Oak represented all coniferous and deciduous trees, respectively [2], [3]. In fact, these two types of tree account for more than two-thirds of all coniferous and deciduous trees in Korea [27], [28]. Previous studies also evaluated this assumption and concluded that it is a reliable approach. More details on the methodologies used in this study are presented in previous studies [2], [3].

### Retrieval of surface phenology from satellite data

The phenological transition dates for vegetation growing seasons were retrieved using the same methodology as in our previous study [8] except that a newly developed plant functional type (PFT) map for South Korea was incorporated to remove non-forest areas, such as irrigated and coastal ocean regions [29], for improved regional accuracy. After removing the non-forest areas, phenological transition dates were calculated for only the temperate forests in such a way that the maximum inflection point of green-up (or down), the date when an individual year's NDVI reached an appropriate threshold value, was chosen as the start of growing season SGS (or end of growing season, EGS), for individual pixels [8]. First, the date *t* with the maximum NDVI_ratio_ ([NDVI(*t*+1)−NDVI(*t*)]/NDVI(*t*)) was determined for the time-averaged NDVI seasonal cycle for 1982–2008. The corresponding NDVI(*t*) was used as the NDVI threshold for the SGS date. The NDVI threshold for the EGS date was determined similarly as the date *t* with the minimum NDVI_ratio_ and the corresponding NDVI at date *t*+1. Using the NDVI thresholds thus obtained, SGS and EGS were calculated for each year. The length of growing season (LGS) was then determined by subtracting SGS from EGS. Here, the time-averaged NDVI seasonal cycle was used to obtain the threshold values in order to remove the effects of transients, such as aerosols, clouds, disturbances, and defoliation, in the annual values [8], [16].

The dates on which the thresholds of SGS and EGS were crossed were calculated from 15-day-resolution NDVI data by interpolating the NDVI data using a 6-degree polynomial regression [8], [16]. To represent NDVI_ratio_(*t*) as a function of days of a year, the 15-day NDVI time-series data from January to September and from July to December were fitted with Julian days using a least-squares regression analysis for the entire study area. After this step, all of the results in this study were based on the daily-resolution annual SGS (and EGS). The use of 6-degree polynomial regression to obtain the daily NDVI from original 15-day-resolution data may not be valid in other regions. In Korea, comparison of the SGS from the interpolated daily NDVI with flowering dates indicates that the 6-degree polynomial regression well captures the daily-scale surface phenology. Based on the three growing season parameters (i.e., SGS, EGS, and LGS) thus obtained, we calculated their spatial and temporal trends. The linear trends for 1982–2008 were calculated by a simple linear regression model with time and the growing season parameters as the independent and dependent variables, respectively.

### Relationship between length of growing season and forest biomass carbon density

To understand the relationship between vegetation growing season and forest biomass carbon stock, we compared the three phenology parameters (SGS, EGS, and LGS) with forest biomass carbon density over the entire 61 sample forests. Because the forest age data were divided into 5 categories (i.e., 10–20, 20–30, 30–40, 40–50, and more than 50 years), we first analyzed the total distribution of age composition over the forests to evaluate the overall age distribution of the entire sample of forests. We then estimated the relationship between forest carbon density and the three phenology parameters.

## Results

### Validation of satellite-retrieved phenology against ground observations

**Figure 1:**
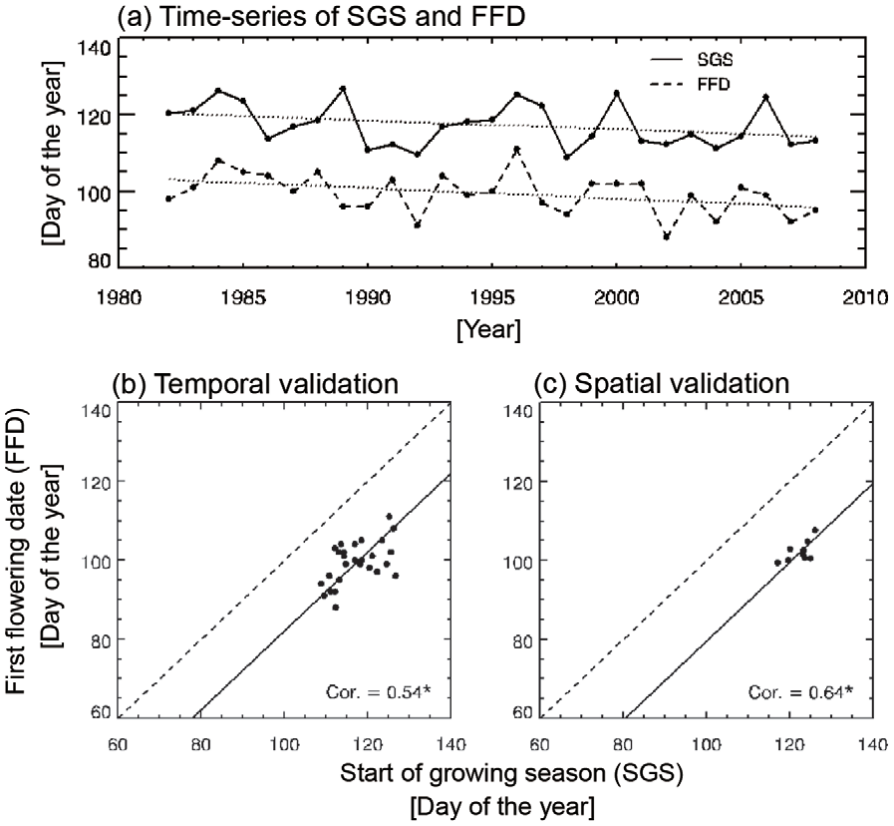
Time series (a) and scatter diagrams (b) for SGS and FFD at Chupungnyeong station for the period 1982–2008.

To validate the satellite-based surface phenology estimates, the relationships between satellite-retrieved SGS and ground-observed FFD were analyzed over 9 stations (Fig. 1). Figure 1a shows time series of SGS and FDD at Chupungryeong station for 1982–2008. During the 27-year period, both spring indices show negative trends of 3.2 days decade^−1^ for SGS and 3.3 days decade^−1^ for FFD. This is consistent with earlier studies on the long-term change in spring phenology in Korea [14], [15]. In terms of interannual variations, the two different phenology events show similar patterns (Fig. 1a) with positive correlations statistically significant at the 95% confidence level (e.g., *r* = 0.53) (Fig. 1b). This indicates that the satellite-retrieved SGS well represents the temporal variations in surface phenology. Strong positive correlations were also observed for the remaining 8 stations (Table 1). The relationship between time-mean SGS and FFD dates at the 9 stations (Fig. 1c) indicates that SGS varies spatially. Each dot in Fig. 1c represents a time-mean value for a station. Both SGS and FFD dates over these 9 randomly distributed stations in South Korea show significant positive correlations, suggesting that the satellite-retrieved SGS reasonably represents the spatial variations in surface phenology.

### Spatial variations in surface phenology

**Figure 2:**
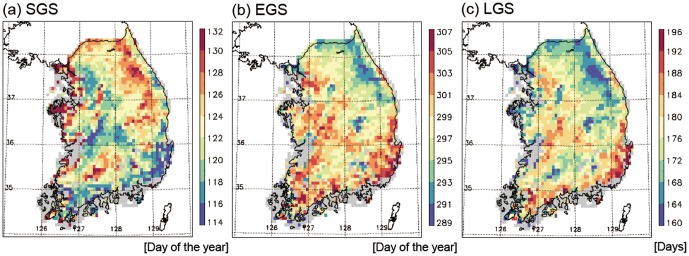
Spatial distributions of averaged SGS (a), EGS (b), and LGS (c) for the period 1982–2008.

**Figure 3:**
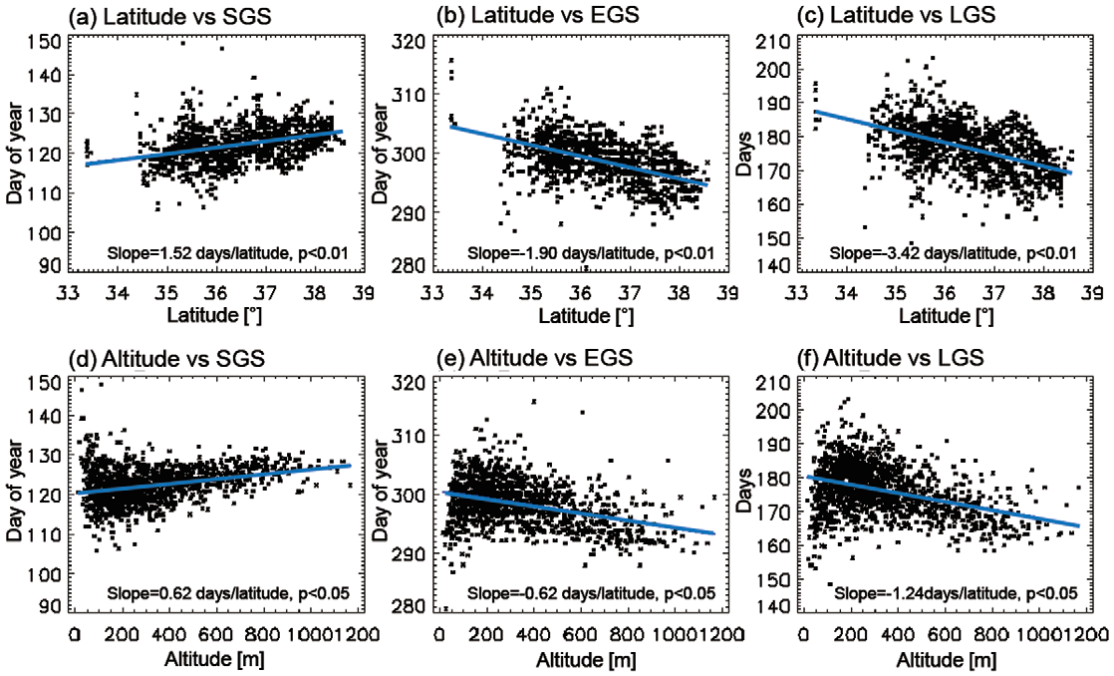
Scatter plots of the three phenology parameters against latitude (a–c) and altitude (d–f) over the analysis regions.

To understand the spatial variations in surface phenology, the spatial patterns of the time-means of the three phenology parameters during 1982–2008 were analyzed (Fig. 2). The SGS dates distributed from day 114 (i.e., 24 April) up to day 132 (i.e., 12 May) (Fig. 2a). Relatively earlier SGS dates (around day 114–116) were predominantly observed in the southern regions around 35°N, and relatively later SGS dates (around day 128–132) were observed in the northern regions around 37°N. The latitudinal gradient is clearly shown by the scatter plot between latitude and SGS (Fig. 3a). Overall, SGS decreased by 1.52 days (degree of latitude)^−1^. The exceptionally late SGS in southern regions around 35.5°N, 127.2°E is attributed to high elevation (>1500 m, Mt. Jiri). Examination of the effects of altitude on the spatial variations in SGS (Fig. 3d) reveals that altitude has considerable effects on SGS variations.

EGS dates ranged from 289 (October 16) to 307 (November 11) (Fig. 2b). The spatial distribution of EGS shows the inverse pattern of SGS, i.e., relatively late EGS dates in the regions of earlier SGS and vice versa. Thus, the latest EGS dates (around day 303–307) were dominant in the southern region along with earlier SGS. EGS decreased by 1.90 days (degree of latitude)^−1^ (Fig. 3b). This result is qualitatively consistent with information on the periods of spatial variations in autumn colors obtained from the Korea Meteorological Agency (http://www.kma.go.kr). The combination of late SGS and early EGS leads to small LGS in northern regions and vice versa (Fig. 2c). For example, the zonal-mean LGS at 37°N is 18% shorter than that at 35°N. The spatial pattern of LGS is generally opposite to the spatial pattern of SGS and similar to the pattern of EGS: longer LGS in the region where the SGS date is relatively early and/or the EGS date is relatively late and vice versa. Thus, the longest LGS (192 to 196 days) is observed in the southern region, and the shortest LGS (around 160–164 days) is shown in the high elevation northeastern region, Mt. Taebaek (>1500 m). The spatial distribution of LGS shows that the time-mean LGS generally decreases with increasing latitude and altitude (Figs. 3c and 3f, respectively).

### Temporal variations in phenology

**Figure 4:**
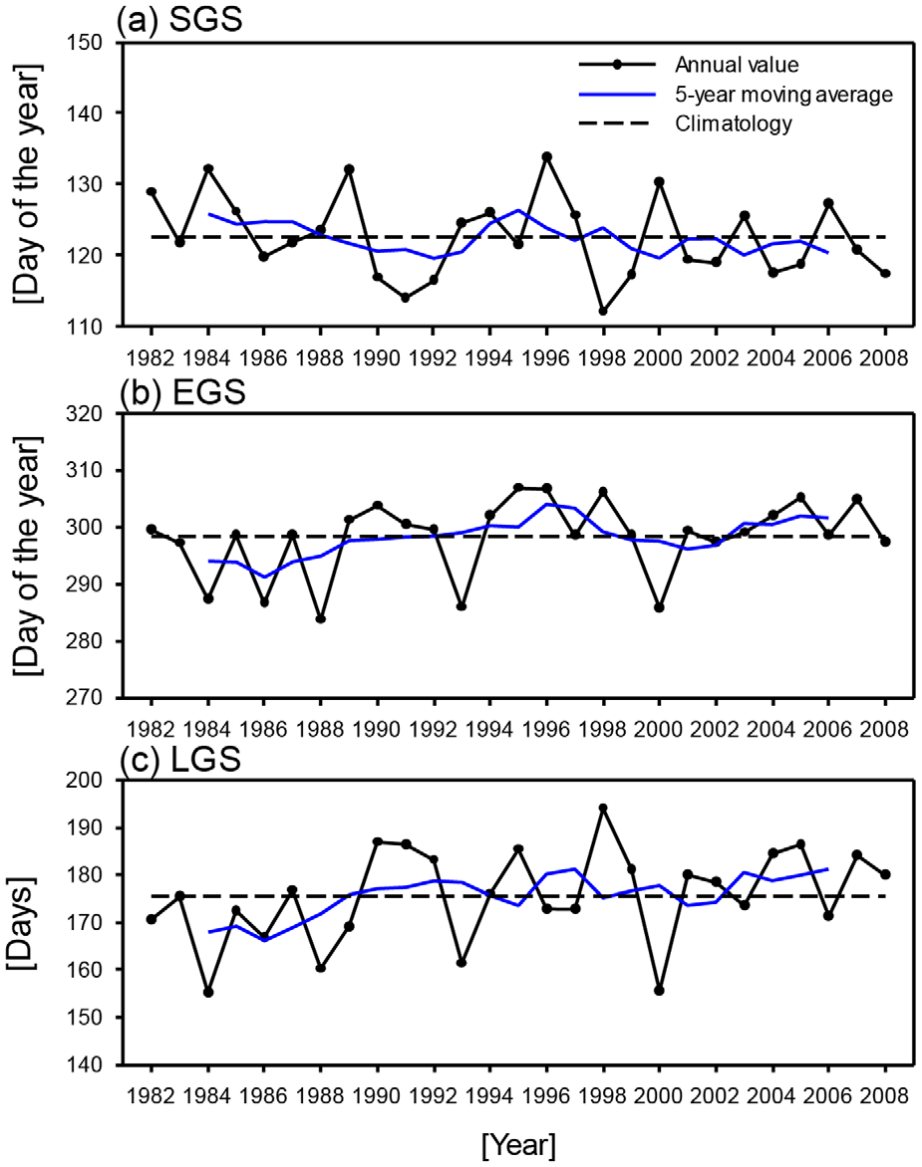
Interannual variations in area-averaged SGS (a), EGS (b), and LGS (c) for the period 1982–2008.

To examine the mean temporal variation in the three phenological parameters (SGS, EGS, and LGS) within South Korea, these parameter values were averaged in the analysis domain. Figure 4 shows the interannual variations in the domain-mean SGS, EGS, and LGS for 1982–2008. As shown in Fig. 4a, SGS shows high interannual variations. Positive anomalies are predominant in the 1980s and negative anomalies are observed in the 1990s and 2000s. Overall, the long-term variations in SGS indicate an “earlier spring” by 1.8 days decade^−1^. In contrast to SGS, negative EGS anomalies are observed in the 1980s and positive anomalies are observed during the 1990s and 2000s, indicating a “delayed autumn” by 2.6 days decade^−1^ (Fig. 4b). The asymmetric long-term variations in SGS and EGS are shown to have led to increases in total LGS of 4.5 days decade^−1^ (Fig. 4c).

**Figure 5:**
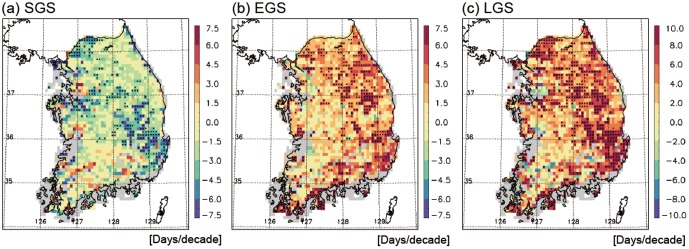
Spatial distributions of linear trends of SGS (a), EGS (b), and LGS (c) for the period 1982–2008. A black dot in the figure indicates regions statistically significant at the 95% confidence level.

To evaluate spatial patterns of the temporal variations in surface phenology, trends of the three phenology parameters during 1982–2008 are presented (Fig. 5). The SGS trend ranges from −7.5 to 7.5 days decade^−1^ (Fig. 5a); the strongly negative trend (around −6.0 days decade^−1^) is randomly observed in the northwestern and southeastern parts of South Korea. In contrast to the SGS trend, EGS shows overall positive trends (Fig. 5b). In most of the analysis region, EGS exhibits trends of 3.5 days decade^−1^. Compared to SGS, EGS shows a more homogeneous spatial distribution. As a consequence of these SGS and EGS trends, LGS shows overall positive trends, indicating lengthening of the growing season (Fig. 5c). Regional variations in the LGS lengthening can be attributed to regional variations in the changes in SGS and EGS. For example, in the northeastern region, positive LGS (8.2 days decade^−1^) is mainly explained by positive EGS trends (5.9 days decade^−1^). In the central region, however, positive LGS (8.3 days decade^−1^) is mainly due to negative SGS trends (−6.1 days decade^−1^). These results suggest that the spatial heterogeneity of vegetation growing season changes cannot be explained by a single phenology parameter alone.

### Relationships between growing season and biomass carbon density

**Figure 6:**
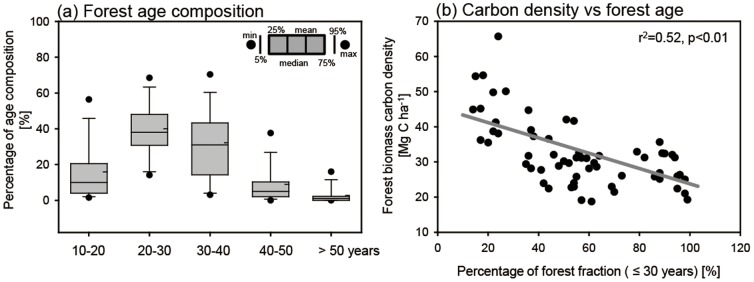
Distribution of forest age composition over 61 forests (a) and scatter plots of forest biomass carbon density against percentage of forest age fractions ≤ 30 years. The gray line denotes the linear regression between the two variables.

Before analyzing the relationship between growing season and biomass carbon density, we checked general features of forest age distribution and biomass carbon density for the entire 61 forests. Figure 6a shows the distribution of forest age composition over the 61 forests. The largest fraction occurs in the 20–30 year category. Overall, most of these forests are relatively young (<30 years) as nationwide reforestation efforts began about 1970 [1]. To understand the relationship between biomass carbon density and forest age composition, we compared carbon density and percentage of forest aged less than 30 years (Fig. 6b). These two variables are negatively correlated (r  = −0.71, significant at the 99% confidence level), indicating that the forests dominated by relatively older trees contain more biomass carbon than do those dominated by younger trees. This is explained mostly by the fact that the magnitude of forest biomass carbon stock increases with succession (i.e., age) or with time from the latest disturbance due to harvest and/or forest fire [30]. The 30-year criterion used here was selected from the inflection point in the distribution of forest age composition for the total forests (e.g., Fig. 6a). However, the negative relationship also occurs if tree-age criteria of 20 years or 10 years are used.

**Figure 7:**
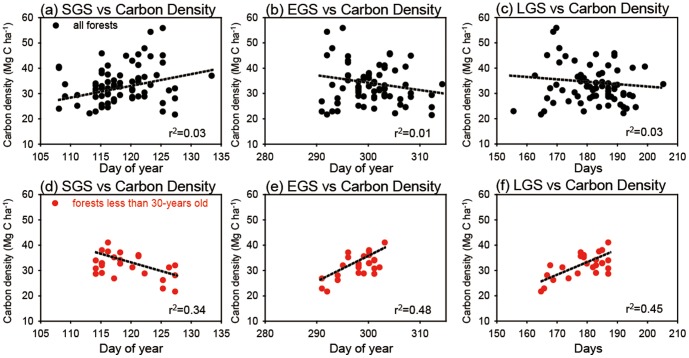
Scatter plots of forest biomass carbon density against the three phenology parameters over all forests (a–c).

Figure 7 shows the relationship between forest biomass carbon density and the three phenology parameters for the entire forests and for the young-tree dominated forests. In the all-forests case, no statistically significant relationships exist between the two variables (Figs. 7a–7c). However, in the young-forest case, statistically significant positive relationships are observed for all three parameters (Figs. 7d–7f), suggesting that the spatial variations in forest biomass carbon density are related with the variations in surface phenology. In addition, of the three phenology parameters, the highest correlation is observed with EGS, indicating that 45% of the spatial variation in forest carbon density in young forests is explained by the EGS variations. These results indicate that the regions of longer growing season have higher biomass carbon density for the young-tree dominated regions.

## Discussion

Satellite-measured vegetation greenness indices (e.g., NDVI, leaf area index, and enhanced vegetation index) from various sensors (e.g., AVHRR, MODIS, and SPOT) have been used widely in land surface phenology studies [8], [16], [17], [22], [31], [32], [33]. It is noted that there are no consensuses about the best datasets and/or methods for evaluating variations in surface phenology [17], [34]. Individual datasets have their own strengths and weaknesses, and the selection of data may depend on the purpose of the study [34]. Among the various combinations of indices and sensors, we used AVHRR-NDVI datasets in this study because of their long-term availability. AVHRR-NDVI has coarser spatial resolutions (e.g., 8 km) than those of MODIS, which may make it more difficult to detect underlying ecological features. To overcome the weakness of satellite-retrieved surface phenology, this study utilized both satellite-based SGS and ground-observed FFD.

A comparison between SGS and FFD showed that FFD was systematically earlier than SGS. Despite the difference, SGS well captured the temporal and spatial variations in FFD at each station and over the entire domain, respectively. The consistent variation between SGS and FFD suggests that satellite-retrieved phenology data can be used for assessing nationwide surface phenology variations in Korea. The absolute difference in the spring dates between SGS and FFD may have been induced by several factors, including the difference in the spatial scales between the two datasets and the species composition within a satellite pixel [18], [34], [35]. Differences in observation periods could also be a potential source of the bias. Relatively coarse temporal resolutions in the raw satellite data (15 days) compared to the ground observations (1 day) may also have contributed to the difference between SGS and FFD [19]. A previous intercomparison study on methods for retrieving surface phenology from satellite remote sensing data reported notable differences according to method [17]. Thus, uncertainties in the retrieval method also contribute to differences between SGS and FFD. One point to note is that the validation of the satellite-based phenology was performed only for a spring phenology parameter because of the lack of ground-observed fall phenology data. More attention to a monitoring system for the final stage of the vegetation growing season (e.g., leaf drop, fall, and colors) is required.

Based on three satellite-retrieved surface phenology parameters, nationwide variations in phenology were evaluated. The three phenology parameters showed clear geographic variations according to latitude and altitude. Temporally, over the entire South Korea domain, LGS increased by 4.5 days decade^−1^ through the last 27 years, mostly due to an increase in EGS dates (2.6 days decade^−1^). Overall, the lengthening of the vegetation growing season further suggests that the nationwide reforestation has responded to the recent climatic warming in South Korea [36]. Although a lengthening of the growing season during the last three decades of the 20^th^ century is consistent with many previous studies [37], [38], [39], [40], most of these focused mainly on the earlier spring contributions to the total growing season. This study identified the prominent role of EGS variations in the growing season. Despite the regional variations in the SGS and EGS trends, the variations in the phenology parameters indicate that enhanced canopy duration is attributed to the delayed leaf drop/color dates. These results are consistent with those of previous studies on surface phenology and ecosystem productivity [31], [33], [41], [42].

Using satellite-retrieved surface phenology data, this study evaluated the relationship between spatial variations in surface phenology and forest biomass carbon density. A statistically significant positive relationship between phenology and biomass carbon density was found in the regions dominated by young forest; however, such a relationship was weak for the forests dominated by old trees. These results indicate that the regions of longer growing season correspond to higher biomass carbon density in the regions dominated by young forest. Owing to limited data, the process behind the positive relationship could not be identified. However, our results may be explained by the simple speculation that spatial variations in biomass carbon density are partially explained by forest growth related to variations in growing season length. This is also supported by a previous study that showed that variations in phenology can modulate forest growth through altering tree diameter at breast height (DBH) in temperate forest regions [43]. This study showed that an enhanced growing season can lead to an increase in DBH and hence increased forest growth. Further, another potential is increased primary productivity through an extended growing season. Because a longer growing season indicates a longer period of taking up growth resources [6], [41], [42], enhanced resources also contribute to the forest growth in the region. In addition, these meaningful relationships are only observed in young-forest dominated regions. This might be explained by the fact that variations in biomass (tree and undergrowth) in young forests are more responsive to external factors (e.g., climatic variations, geography, natural disturbances, and land cover) than are those in old forests [30], [44]. Therefore, although old-forest dominated regions might be related to growing season, it is difficult to detect the relationship in the present study. Overall, these results indicate that changes in vegetation growing season should be included in large-scale forest management.

An important thing to note is that this study does not suggest that surface phenology is the only factor explaining forest biomass carbon stock. In general, it is well known that forest biomass carbon stock is explained mostly by forest age, composition, structure, scale, location, and the nutrient cycle of the forest [30], whereas the contribution of surface phenology has not been sufficiently addressed. This absence of surface phenology is also seen in previous studies on the evaluation of factors affecting variations in forest carbon stock in the same region and with the same data used in the present study [2], [3]. Therefore, the observed positive relationship in this study suggests that variations in surface phenology are also related with the variations in forest biomass carbon stock. Among the three surface phenology parameters, the highest correlations occurred with EGS. This is consistent with previous studies on ecosystem-level vegetation primary production [41], [42] that showed that enhanced net primary production (or carbon uptake) could be attributed to delayed fall phenology (or the time for carbon exchange in fall) [41], [42]. Besides, Wu et al. [42] found that an autumn event (e.g., cessation of carbon uptake) is a much better index for explaining net ecosystem productivity, implicating the importance of autumn phenology.

Because our carbon density data are based on vegetative biomass, we did not consider the soil component in the forest ecosystem. The general consensus on this relationship is that an increase in canopy duration leads to an increase in the carbon uptake period [6]. However, the exact amount of carbon uptake is regulated by the relative dominance between gross primary production and ecosystem respiration [10]. Thus, to further understand the exact role of phenology in the terrestrial carbon pool (or exchange), we need more systematic evaluations with soil dynamics. Besides, other factors (e.g., species composition, height or diameter class, understory vegetation, and secondary vegetation in the forest) can also influence the relationships between phenology and the carbon cycle to some extent. Finally, additional mechanical analyses should be done by taking into account local measurements (e.g., flux network and ground phenology data), forest inventory databases, satellite data, and terrestrial ecosystem models.
